# Effects of climate change on a mutualistic coastal species: Recovery from typhoon damages and risks of population erosion

**DOI:** 10.1371/journal.pone.0186763

**Published:** 2017-10-26

**Authors:** Yu-Ting Chiu, Anthony Bain, Shu-Lin Deng, Yi-Chiao Ho, Wen-Hsuan Chen, Hsy-Yu Tzeng

**Affiliations:** 1 Department of Forestry, National Chung- Hsing University, Taichung, Taiwan; 2 Institute of Ecology and Evolutionary Biology, College of Life Sciences, National Taiwan University, Taipei, Taiwan; 3 Chungpu Research Center, Forestry Research Institute, Chiayi, Taiwan; Ecole Polytechnical university, CHINA

## Abstract

Presently, climate change has increased the frequency of extreme meteorological events such as tropical cyclones. In the western Pacific basin, these cyclones are called typhoons, and in this area, around Taiwan Island, their frequency has almost doubled since 2000. When approaching landmasses, typhoons have devastating effects on coastal vegetation. The increased frequency of these events has challenged the survival of coastal plant species and their posttyphoon recovery. In this study, a population of coastal gynodioecious *Ficus pedunculosa* var. *mearnsii* (Mearns fig) was surveyed for two years to investigate its recovery after Typhoon Morakot, which occurred in August 2009. Similar to all the *Ficus* species, the Mearns fig has an obligate mutualistic association with pollinating fig wasp species, which requires syconia (the closed *Ficus* inflorescence) to complete its life cycle. Moreover, male gynodioecious fig species produces both pollen and pollen vectors, whereas the female counterpart produces only seeds. The recovery of the Mearns fig was observed to be rapid, with the production of both leaves and syconia. The syconium:leaf ratio was greater for male trees than for female trees, indicating the importance of syconium production for the wasp survival. Pollinating wasps live for approximately 1 day; therefore, receptive syconia are crucial. Every typhoon season, few typhoons pass by the coasts where the Mearns fig grows, destroying all the leaves and syconia. In this paper, we highlight the potential diminution of the fig population that can lead to the extinction of the mutualistic pair of species. The effects of climate change on coastal species warrant wider surveys.

## Introduction

Current climate changes induce markedly varying environmental changes [1], which increasingly affect ecosystems [2]. One of the most pronounced changes in the climate patterns is the increased frequency and intensity of tropical cyclones [3,4], with a striking example being Taiwan, which experienced an average of 3.3 cyclones (called typhoons in the northwestern Pacific basin) annually before 2000, thereafter increasing to 5.7 typhoons annually [5]. Tropical cyclones markedly affect coastal environments by facilitating invasive species spread and indirectly increased the pressure on natural environments from human populations [6] as well as by reducing species biodiversity [7]; overall, the amount of damage increases with the intensity and the frequency of tropical cyclones [8]. Typhoons play a critical role in the reproduction, regeneration, and succession of trees in tropical and subtropical forests [9,10]. However, recently, global warming-induced extreme climate change has increased the frequency of severe typhoons that bring heavy rains and strong winds [1], thus considerably affecting the stability of forest ecosystems [6,7].

The global average surface temperature has increased by 0.74°C ± 0.18°C in the past 100 years (1906–2005) [1], with the current global record exceeding the series of monthly temperatures [11]. Increased temperatures first cause a shift in the species distribution [2] and many more subtle changes in ecosystems. Among them, the effects on plant phenology involve an earlier flowering onset and a gap in flowering events in the middle of the year [12,13]. In concordance with the increased typhoon frequency, a meteorological data analysis revealed that during the past century, the average warming trend was stronger in Taiwan than that worldwide; between 1911 and 2009, the annual mean temperature increased by 1.4°C, which is equivalent to a warming rate of 0.14°C per decade, and for the past 30 years (1980–2009), it increased by 0.29°C per decade, which is more than twice the average rate in the past century [14,15].

Plant phenology explores the relationship between seasonal meteorological procedures and plant activities such as leaf flushing and fruiting [16]. Because plant phenology is associated with climatic factors [17], the current global changes in the meteorological patterns are challenging research on plant phenology. Since the end of the last century, the number of studies on phenology and climate change published annually has increased almost 10 times [16]. Plant phenology is complex, and many reviews have provided a synthetic and potentially novel perspective on phenological studies conducted under climate change [16,18,19]. Although temperature can drastically affect plant phenology [20], precipitation is extremely important in many ecosystems [21,22]. One of the major modifications of climate change is the disturbance of the global precipitation pattern [23], with severe droughts occurring more frequently [24]. A similar pattern was observed in the extreme south of Taiwan: less rain and increased drought frequency and temperatures [25]. Moreover, the shoreline of the Hengchun Peninsula forming the southern tip of Taiwan Island is partly composed of uplifted coral reef remnants reaching a maximum altitude of 10 m. The coral remnants are sharp and dark rocks, where very few plant species can grow, including two *Ficus* species: *Ficus tinctoria* subsp. *swinhoei* (Swinhoe fig) and *F*. *pedunculosa* var. *mearnsii* (Mearns fig). The Mearns fig exclusively grows on the uplifted coral reef remnants.

*Ficus* (Moraceae) is one of the most diverse genera of woody plants, with approximately 750 pantropical species divided almost equally between two breeding systems: monoecy or functional dioecy [26]. The characteristic of the genus is an enclosed inflorescence called syconium or fig. Fig tree populations produce syconia throughout the year [27], which make them a critical food resource for frugivores, particularly during the food scarcity season [28]. Thus, they are considered one of the keystone species in tropical forests [29,30]. Furthermore, the *Ficus* genus is known for its obligate mutualistic relationship with pollinating wasps, which is often considered an extreme example of plant–animal coevolution [31]. Each fig species is typically pollinated by females of a single species-specific agaonid wasp (but see [32,33]), consequently providing oviposition sites for the pollinating wasp and food for its larvae. The short-lived pollen-carrying female wasps emerge from ripe figs and locate other receptive figs to lay eggs [34]. The asymmetry between the lifespans of the pollinating wasp (a maximum of few days [35, 36]) and fig tree is a strong selective pressure for the mutualism. A pollinating fig wasp must find a receptive fig within a few hours of its birth; therefore, the associated *Ficus* species must provide oviposition sites. Indeed, fig tree populations have often been reported to be highly asynchronous and produce figs throughout the year [27,37]. When catastrophic climate events occur, such as droughts, fig trees cannot produce syconia; therefore, the local population of wasps becomes extinct and the recovery is long [38].

The uplifted coral reef remnant shore in the extreme south of Taiwan is threatened by climate change. Indeed, local species grow in a harsh environment (hot and salty, with frequent droughts and low nutrient availability) at a very low altitude (less than 10 m) and the threat posed by sea-level rise and abnormal weather by climate change is real [4]. Therefore, Taiwan’s seashore can be categorized as vulnerable [9]. Typhoons considerably affect terrestrial ecosystems; therefore, the recovery ability of coastal plants after major meteorological disturbances must be investigated. For example, Typhoon Morakot (August 2009) induced the withering of most aboveground vegetation in coastal areas. *Ficus pedunculosa* var. *mearnsii* is probably well adapted to its environment as it colonized the uplifted coral reef remnant shore in the south of Taiwan. Nevertheless, this environment is considered harsh for the Mearns fig because it is part of an obligate mutualism and, to the contrary to the other local species that can produce flowers and fruits when the conditions are favorable, the Mearns fig trees have to produce figs to maintain their pollinating fig wasp population. Thus, the studied environment was considered as harsh for a *Ficus* species.

Regarding obligate mutualisms (Mearns fig and its associated pollinated wasp species), the damage engendered by typhoons is unknown. In this study, we focused on the Mearns fig population in the Hengchun Peninsula in the south of Taiwan after the occurrence of Typhoon Morakot. The phenology of both leaf and syconium abundance was observed over a 2-year survey period. This study was conducted to determine (1) the phenology of the Mearns fig immediately after the disturbance, (2) whether the phenology showed sexual differentiation during the recovery period, (3) the syconium reproductive strategy of male and female trees, and (4) the typhoon-induced damage in the mutualism. Our study explored the adaptive strategy of the Mearns fig in the harsh environment of coastal uplifted coral reef remnants.

## Material and methods

### Species and sites

**Fig 1:**
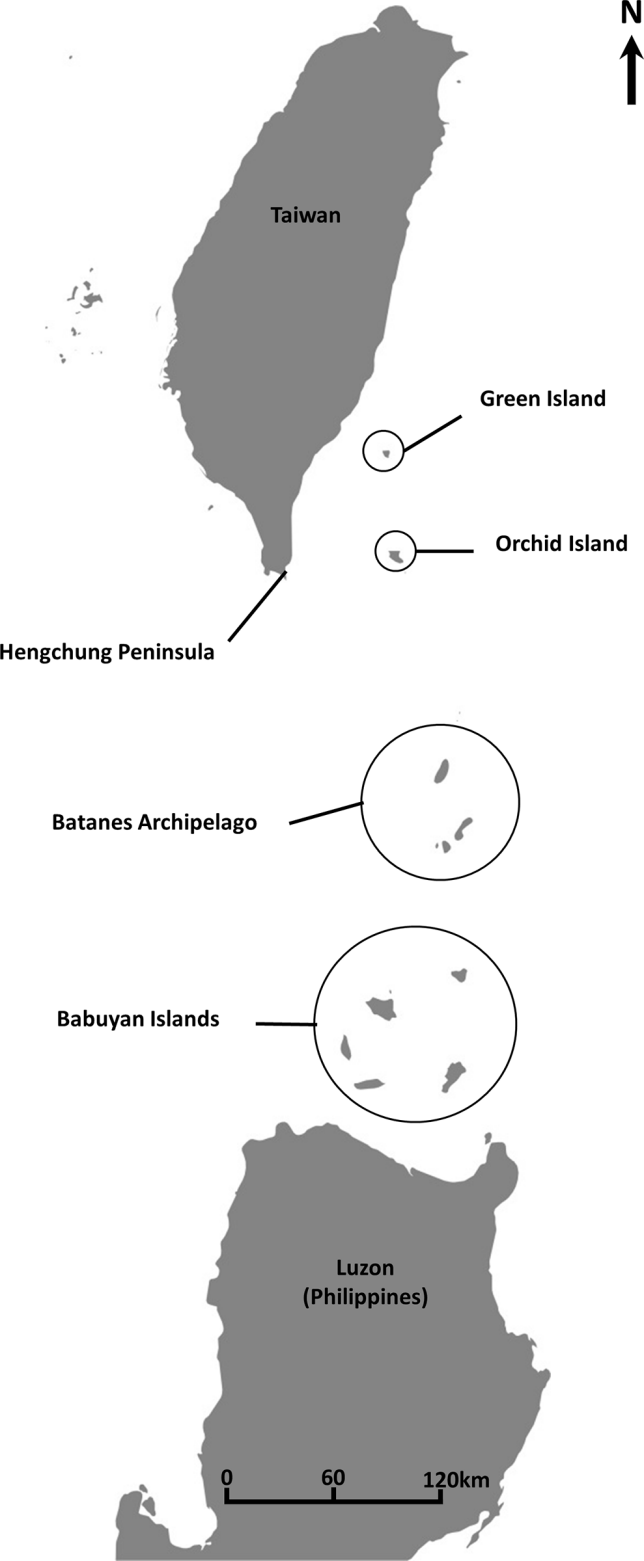
Distribution area of the Mearns fig (Taiwan islands and Northern Philippines). The map is comprised in these coordinates: 25°18’N; 119°19’E and 15°54’N; 122°32’E.

**Fig 2:**
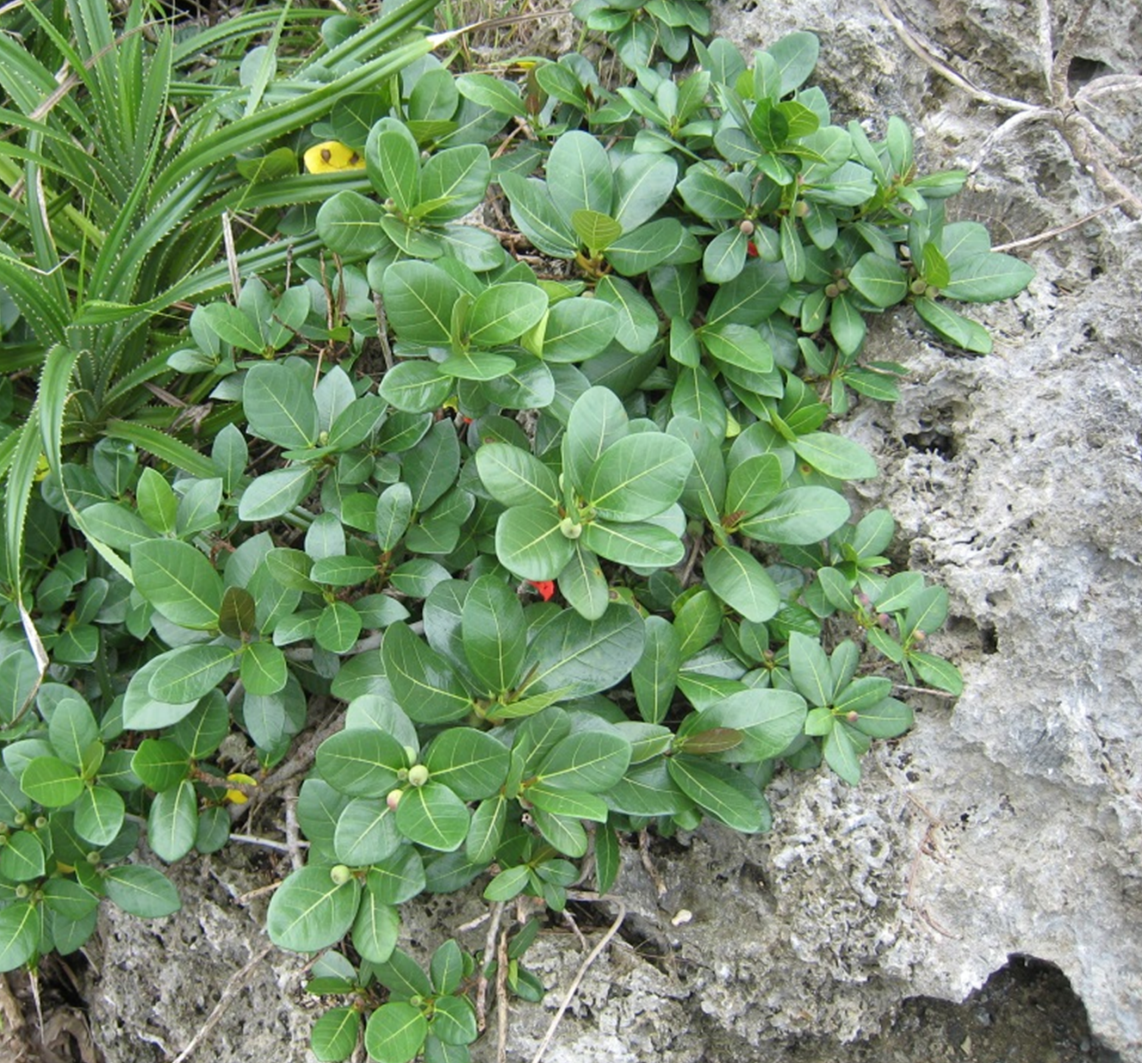
*Ficus pedunculosa* var. *mearnsii* on the Frog Rock Trail, Hengchun peninsula, South Taiwan.

*Ficus pedunculosa* Miq. var. *mearnsii* (Merr.) Corner (or Mearns fig), a rare fig variety, is distributed only in the south of Taiwan Island [39, 40]: Orchid and Green Islands in Taiwan, as well as in Luzon, Babuyan Islands, and the Batanes archipelago in the Philippines [39,41,42]. Therefore, Taiwan is the northern boundary of the Mearns fig distribution (Fig 1). The Mearns fig, a deciduous dioecious fig, belongs to the section *Ficus* of the subgenus *Ficus* [41]. It is a small shrub, approximately 0.3–1 m high, living in the crevices of uplifted coral reef remnants (Fig 2). A pollinating wasp, *Blastophaga pedunculosae*, and a nonpollinating fig wasp, *Apocrypta* sp., are associated with Mearns figs [42,43]. *Ficus pedunculosa* var. *mearnsii* is not an protected species under the Taiwan law.

According to Galil and Eisikowitch, syconium maturation occurs in five phases [44]: prefemale phase (A phase), female phase (B phase, receptive phase), interfloral phase (C phase), male phase (D phase), and mature phase (E phase). During the B phase, the female pollinating wasp enter the fig to pollinate the receptive female flowers inside. Then, the wasp larvae in male figs and the seeds in female figs mature during the C phase. At the end of the C phase, the wasps hatch, mate and exit the figs (D phase) or the figs ripen (E phase). The sexual functions of dioecious figs vary in trees; therefore, female figs have no D-phase figs and male figs have no E-phase figs.

In the Hengchun Peninsula, the southernmost part of Taiwan Island, the coastline is constituted by uplifted coral reef remnants. The study site was located along the Frog Rock Trail (21°56ʹN, 120°48ʹE; altitude, 2–10 m) in Kenting National Park (Fig 1). The research permit was issued by Mr. Qing Lin, director of the Kenting National Park in 2009.

The annual average accumulative rainfall and average temperature are 2,022.4 mm and 25.1°C, respectively (1980–2010, Central Weather Bureau). The climate is seasonal; most of the rainfall (91%) occurs during the warm–wet season and the typhoon season occurs from May to October [45], and it is considered to be equatorial and monsoonal [46]. Besides the study species, the local coastal vegetation is composed mainly of the following species: *Premna serratifolia*, *Hibiscus tiliaceus*, *Pemphis acidula*, *F*. *tinctoria* ssp. *swinhoei*, *Sophora tomentosa*, and *Clerodendrum inerme*.

### Phenological survey

The phenological survey of the Mearns fig was conducted from September 2009 to August 2011 at an interval of 10–22 days (average, 14.3 days). A total of 62 trees (33 and 29 male and female trees, respectively) were monitored, but four male and eight female trees died during the survey. Each tree had 2–10 marked branches of approximately 20 cm length: as the trees are tiny shrubs some individuals had few long enough branches. At each survey, the syconium abundance and developmental phases, as well as the number and category of the leaves (young, mature, or senescent), were recorded. A crop is defined by the production of figs from A-phase to the final phase of development. Thus, if a individual produces new figs over a long period it will have a long crop. On the other hand, if the A-phase production is interrupted two crops can overlap (see S1 Fig in supplementary material).

### Data analysis

A crop was defined as the succession of syconia from phase A to the final developmental phase. Trees can produce a new crop before the previous crop has completely developed; the new crop was noted as an overlapping crop. Furthermore, we defined seasons as spring (March–May), summer (June–August), fall (September–November), and winter (December–February).

We tested the possible correlations between the production of syconia and number of leaves in each survey by using the Kendall rank correlation test, as well as the meteorological factors (temperature and rainfall) and *Ficus* phenology [47]. Subsequently, we calculated the ratio of the number of syconia and mature leaves on each branch (referred to as the fig:leaf ratio) to explore the relationship between syconium production and photosynthesis. The average fig:leaf ratio was then calculated for each tree. We used the Kolmogorov–Smirnov Z test to compare the phenology of the figs and leaves and the fig:leaf ratio between male and female trees. All statistical analyses were performed using SPSS 12.0 (IBM SPSS Statistics, Chicago, IL, USA).

## Results

### Postdisturbance phenology

**Fig 3:**
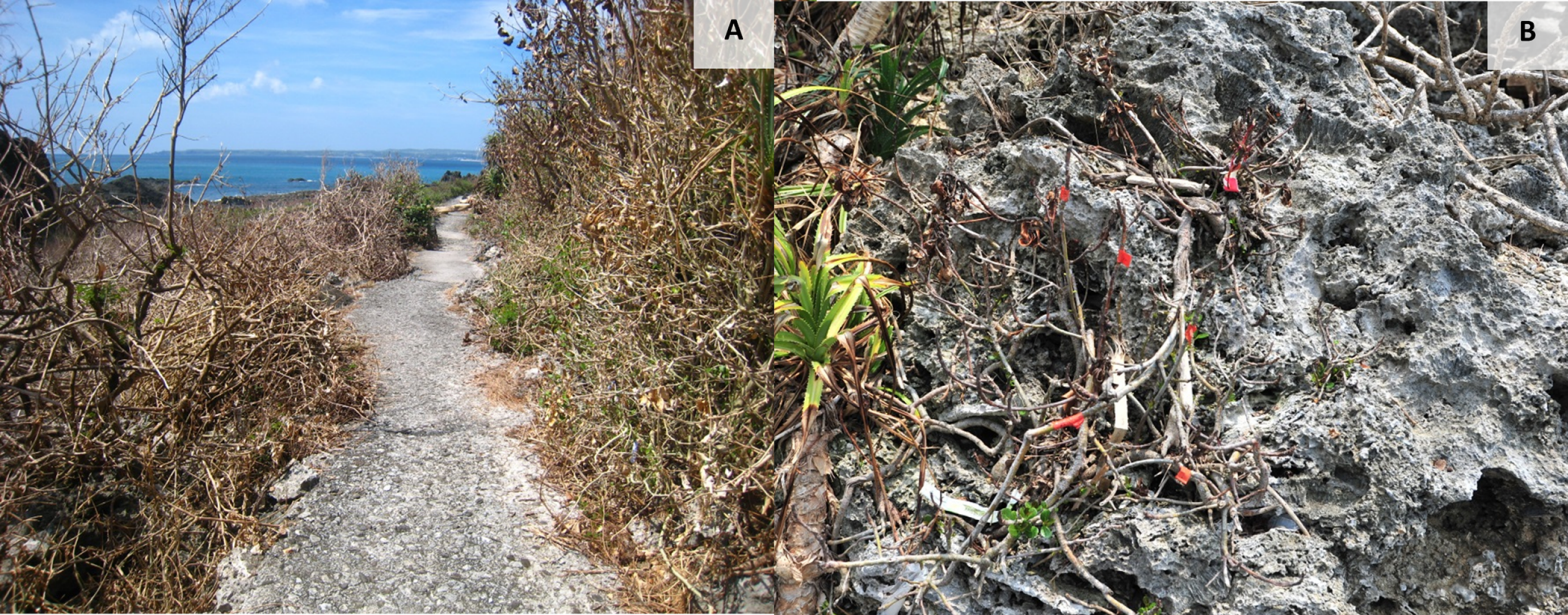
The Frog Rock Trail vegetation after the Typhoon Morakot. Overview of the vegetation (A); One *Ficus pedunculosa* var. *mearnsii* individual (same individual on the Fig 2) after the Typhoon Morakot (B).

After Typhoon Morakot in the Hengchun Peninsula, all the Mearns fig trees as well as many other plants on the Frog Rock Trail were almost entirely withered (Fig 3). In other words, all the pollinated figs were lost with the generation of larva growing into them. Only a few individuals that lived in sheltered rock cavities retained some nonwithered branches with C phase figs.

**Fig 4:**
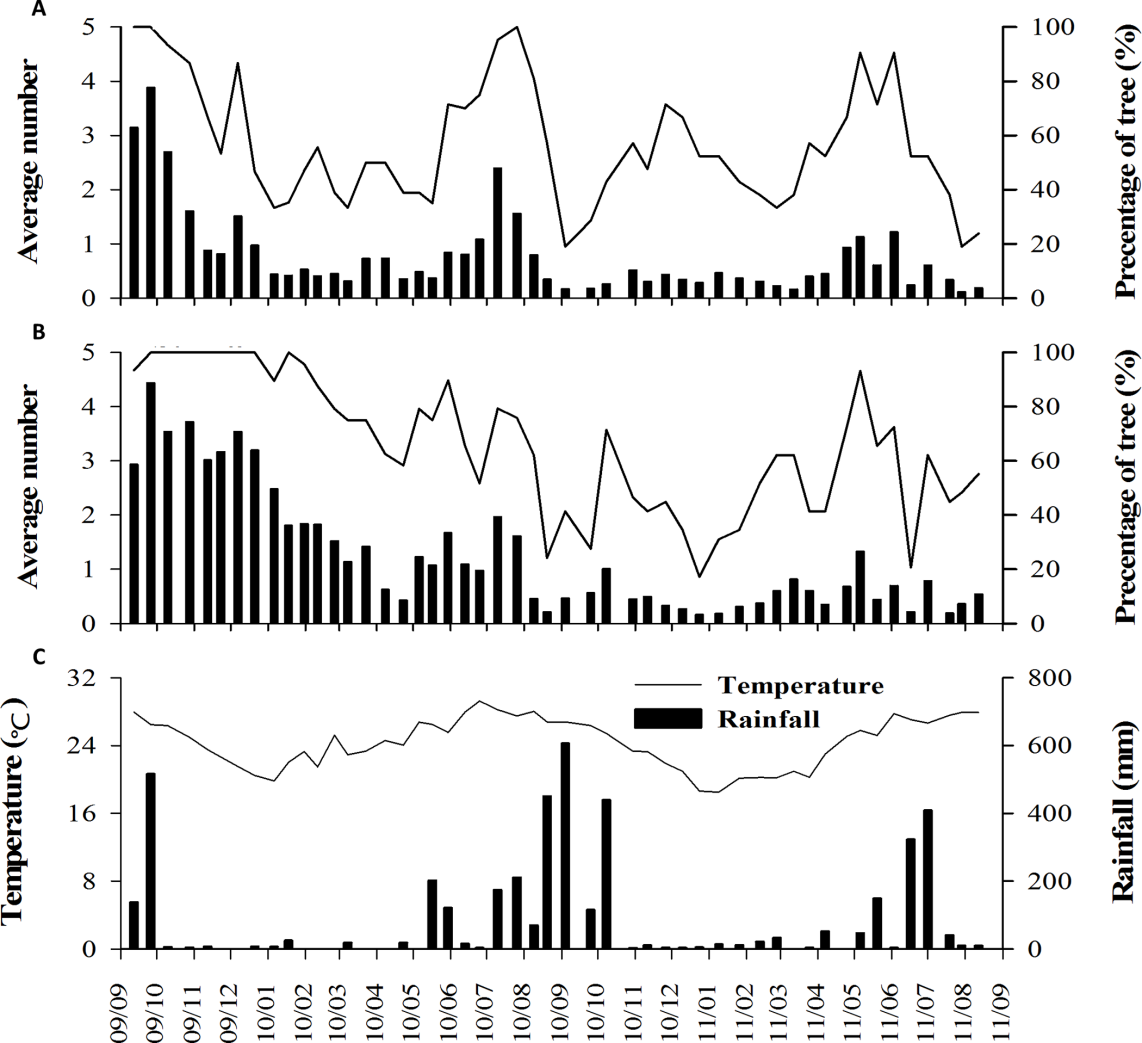
Syconium production onset and meteorological factors. A-phase syconium production (average number per branch) for female (A) and male (B) trees during the survey period and the meteorological factors during the same period (C): September 2009 to August 2011. The bars on Fig 4A and 4B represent the average number of A-phase syconia and represent the weekly rainfall on Fig 4C. The lines on Fig 4A and 4B represent the percentage of trees bearing A-phase figs and represent the average weekly temperature on Fig 4C.

**Fig 5:**
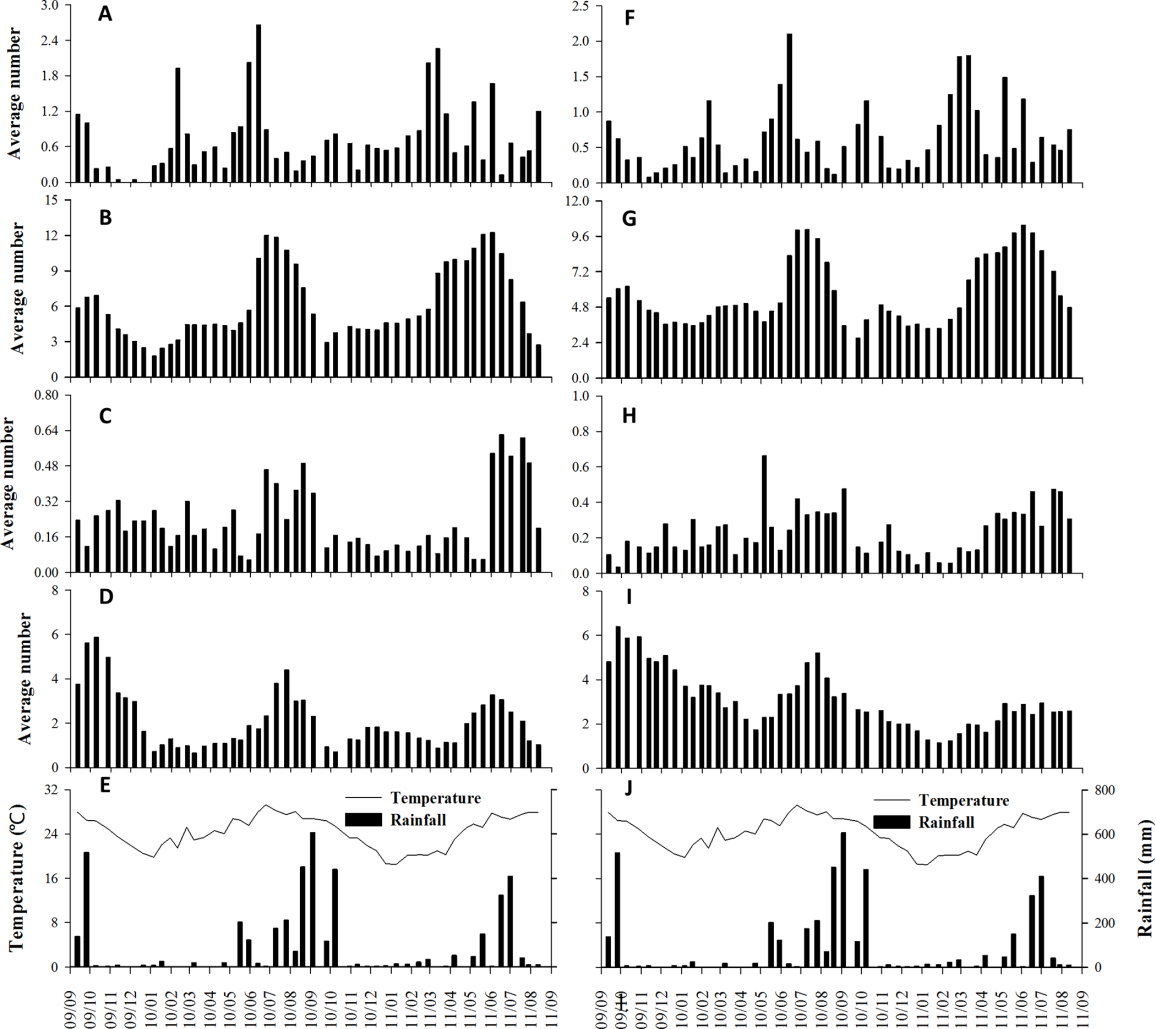
Average number per branch of the different leaf and syconium phases. Young (A), mature (B) and senescent (C) female leaves and total female syconia (D); Young (F), mature (G) and senescent (H) male leaves and total male syconia (I) during the survey period; and the meteorological factors during the same period (E, J): September 2009 to August 2011.

**Fig 6:**
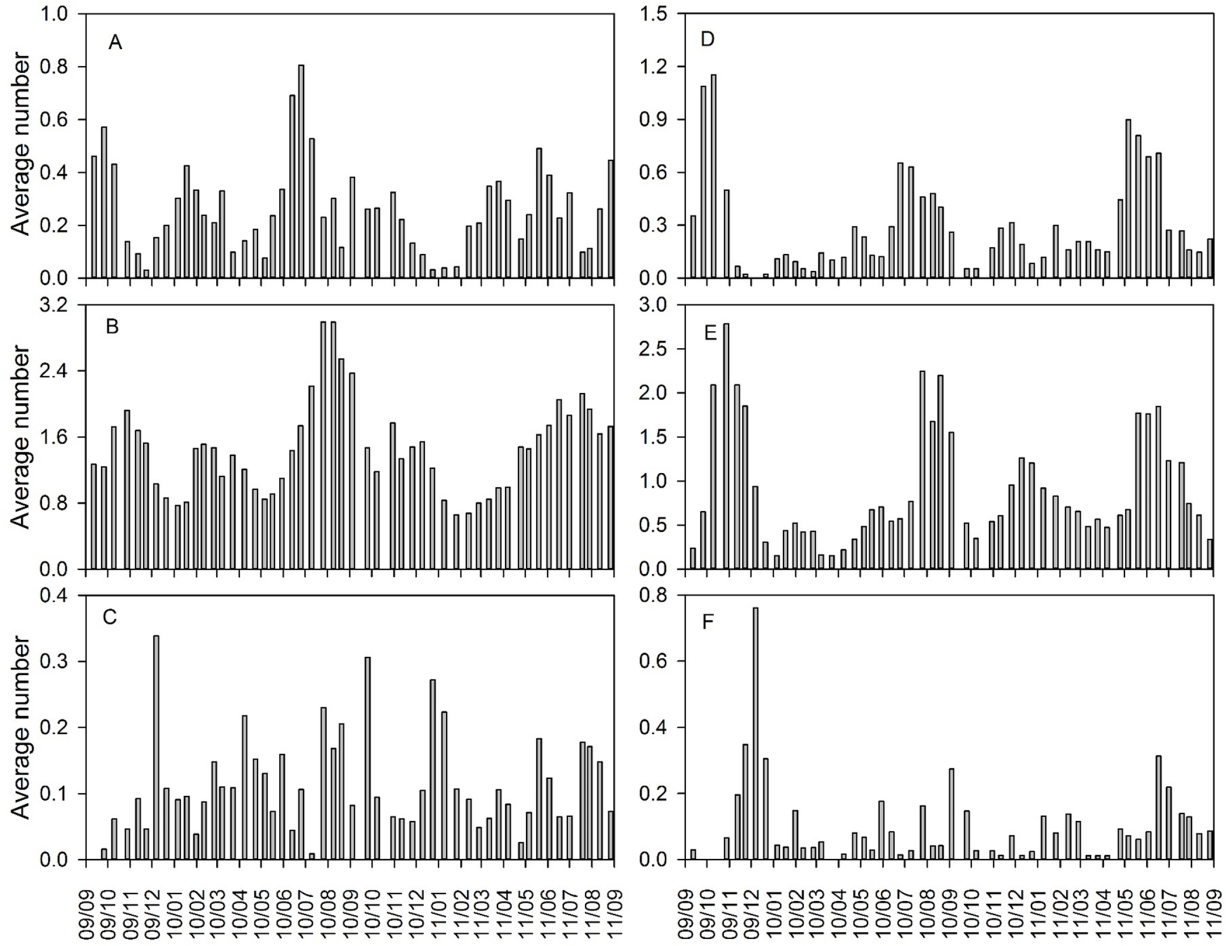
Average number per branch of B-, C-, D-, and E-phase syconia. Male B- (A), C- (B), and D- phase syconium (C) and female B- (D), C- (E), and E-phase syconium (F) during the survey period.

The phenological recovery of Mearns figs was rapid. Within the initial months after the disturbance, the average number of A-phase syconia was the highest for both male and female trees (Fig 4). The trees also produced new branches and foliages (Fig 5A and 5F). Moreover, the number of male trees bearing A-phase syconia was the highest at the beginning of the survey (Fig 4). The syconia were quickly pollinated: the number of C-phase syconia increased few weeks after the typhoon (Fig 6).

Three main A-phase syconium production periods (i.e., crop onsets) were defined in female trees: September 2009 to January 2010, June–August 2010, and April–June 2011 (Fig 4A). Each of these production periods was shorter and less fruitful than the previous period. Contrastingly, the production peaks in male trees occurred between September 2009 and March 2010 and May–August 2010, with two additional production peaks in October 2010 and May 2011 (Fig 4B). The abundance of A-phase syconia and number of trees producing A-phase syconia did not significantly correlate with the meteorological factors (average temperature and rainfall) for both male and female trees (Table 1).

**Table 1:** Kendall’s rank correlations (τ) between climate and phenological factors.

	Temperature		Rainfalls	
	Male	Female	Male	Female
Fig				
A phase	NS	NS	NS	NS
Fig total number	0.306**	0.277**	NS	NS
A%	NS	NS	NS	NS
Leaf				
Tender	NS	NS	NS	NS
Mature	0.447***	0.321**	NS	0.246*
Falling	0.441***	0.343***	NS	NS
Fig/Leaf ratio	NS	NS	NS	NS

A% represent the proportion of trees bearing syconia. NS: Not significant

* P<0.05

** P<0.01

*** P<0.001

### Phenology

Leaf production and falling occurred throughout the year. After Typhoon Morakot, the trees recovered foliage; both male and female trees had a leaf production peak at the beginning of March 2010, followed by another peak in June (Fig 5A and 5F). This pattern was repeated in 2011. Thus, the number of mature leaves peaked in summer for both male and female trees (Fig 5B and 5G). The abundance of senescent leaves increased shortly after the peak abundance of mature leaves (Fig 5C and 5H). Although the Mearns fig is a deciduous species, the period without leaves is short for each tree, and the trees produce leaves throughout the year. The production of leaves and number of senescent leaves were similar between male and female trees (S1 Table). According to the Kendall correlation results, leaf and fig production did not correlate with any of the meteorological factors (Table 1). By contrast, temperature correlated with many phenological characteristics, whereas rainfall only correlated with the number of female mature leaves (Table 1).

Mearns fig trees produced figs throughout the year (Fig 5D and 5I). A total of 158 crops were recorded from 29 male trees [average: 5.2 ± 1.2 (mean ± standard deviation), range: 3–7], whereas 90 crops were produced by 21 female trees (average: 4.7 ± 1.0, range: 2–7; S1 Fig). The number of crops yielded by male and female trees was not significantly different (Mann–Whitney U test; U = 224 and p = 0.102), as was the total number of figs; however, the number of C-phase figs was significantly greater in male trees than in female trees (S1 Table for details). Mearns fig trees produced figs throughout the year, but with clear seasonality (Fig 4). The largest trees yielded figs throughout the year, but the small trees produced figs discontinuously (S1 Fig).

### Relationship between fig and leaf phenology

**Fig 7:**
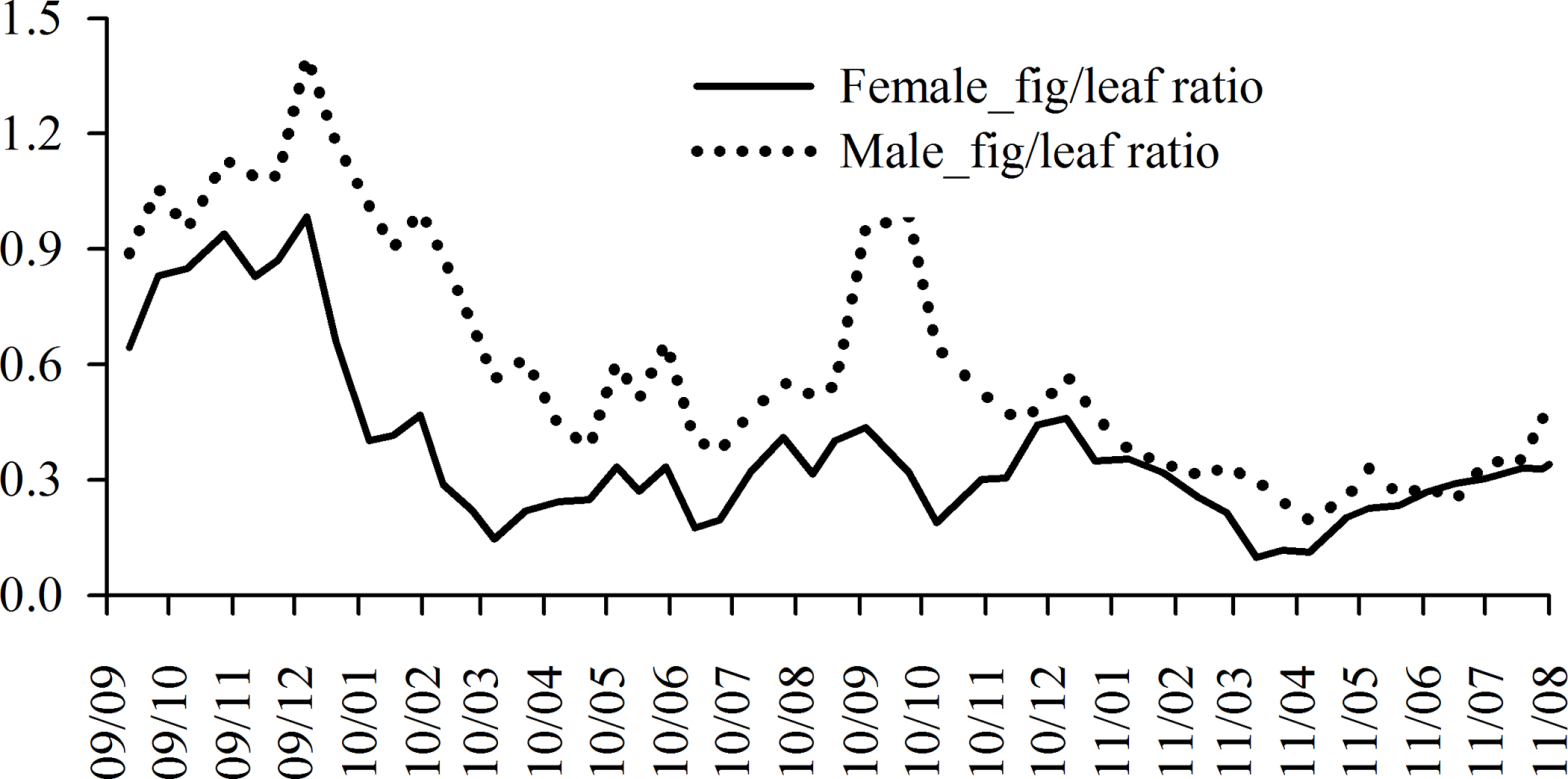
Fig/leaf ratio of *Ficus pedunculosa* var. *mearnsii* for both genders in Frog Rock Trail from September 2009 to August 2011.

For both male and female trees, the fig:leaf ratio peaked within four months after Typhoon Morakot (Fig 7). From six months after Typhoon Morakot, the fig:leaf ratio for female trees remained constant at nearly 0.3 during the entire survey. Contrastingly, the aforementioned ratio showed higher variations for male trees, with an increase at the beginning of of the autumn of 2010. The average fig:leaf ratio for male trees (5.7) was significantly higher than that for female trees (3.7; S1 Table); meteorological factors and the fig:leaf ratio were not related in both male and female trees (Table 2).

**Table 2:** Kendall’s rank correlations (τ) between the leaf and fig phenology. Significant p-values are indicated in bold.

		Female			Male		
		Tender leaf	Mature leaf	Falling leaf	Tender leaf	Mature leaf	Falling leaf
A phase	τ	-0.060	0.200	0.093	0.057	0.029	-0.110
	ρ	0.541	**0.040**	0.340	0.558	0.763	0.262
Total syconia	τ	-0.179	0.362	0.179	-0.092	0.128	0.071
	ρ	0.067	**0.000**	0.068	0.345	0.189	0.467

The abundance of female mature leaves was positively and significantly correlated with both A-phase and total female figs, but no additional significant correlation was found for female and male phenological factors (Table 2).

## Discussion

### Risks on Mearns fig population

In Taiwan, the Mearns fig population grows on the shoreline up to an altitude of 10 m. The number of individuals on Taiwan Island was less than 2000 in 2010 (Chiu & Tzeng, unpublished data), and it was estimated to be nearly 200 on Orchid and Green Islands collectively. The Mearns fig population in Babuyan and Batanes Islands can be very roughly estimated between 2000 and 3000 individuals.

**Fig 8:**
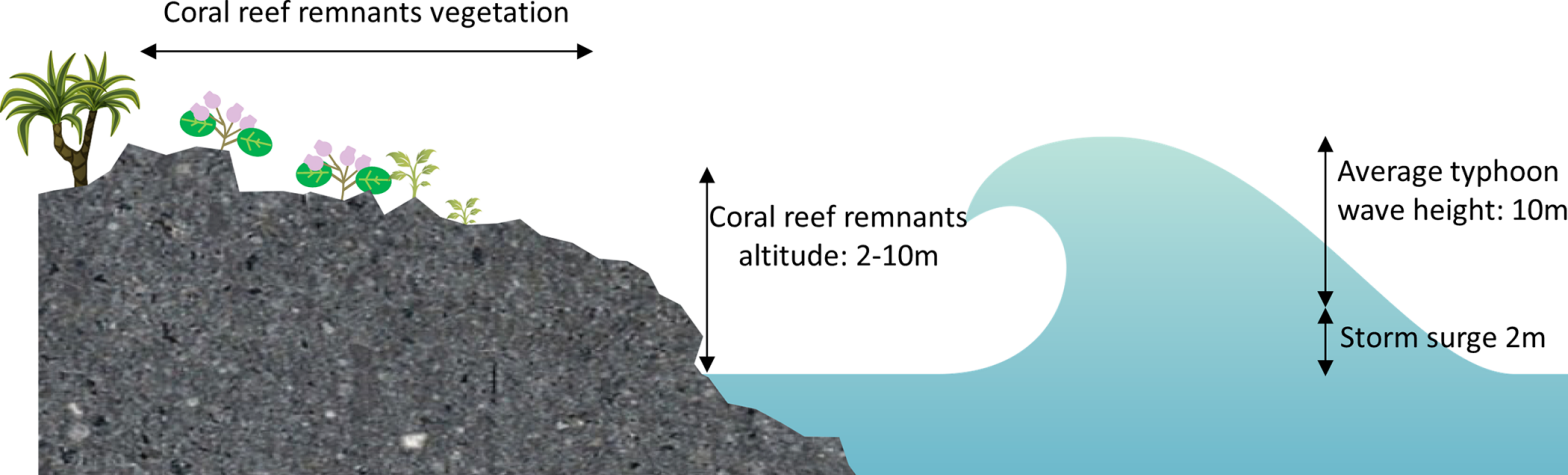
Profile of the coral reef remnant coastline during typhoon.

Typhoon waves exert devastating effects on coastal species. If these waves reach the vegetation, the direct kinetic effects and the effects of salty water can completely wither the coastal vegetation, with no recovery. For example, the waves of Typhoon Morakot reached a height of 11 m near the study site [48]; moreover, waves with a height of more than 15 m were observed during Typhoon Krosa in 2007 on the east coast of Taiwan [49]. However, most typhoons that passed Taiwan between 1995 and 1998 produced 8-m high waves [50]. Moreover, typhoons momentarily increase the sea level. This phenomenon is called storm surge and can increase the sea level by up to 2 m [51]. Considering the proximity of the Mearns fig population to the coastline and its elevation, any typhoon passing by the southern peninsula of Taiwan Island can be considered to result in disastrous consequences, possibly withering all the trees (Fig 8). Approximately 44% of the typhoons passing by Taiwan before 1999 passed by the Hengchun Peninsula [50]. Thus, as reported by Tu et al. [5], 2.5 typhoons can be estimated to affect the Mearns fig population annually.

Typhoons reduce the temperature and humidity in Southern Taiwan because of adiabatic warming (or foehn wind) on crossing the Central Mountain Range in Taiwan [52]. This phenomenon was strong during Typhoon Morakot [53]. Moreover, droughts in Southern Taiwan have markedly increased in magnitude and duration since the second half of the twentieth century, and the recent drought in 2002 lasted 13 months [25].

### Phenological recovery and mutualism

The passage of Typhoon Morakot over the Hengchun Peninsula has resulted in the same damages, such as defoliation and withering, as have many other tropical cyclones [54,55]. Studies on postcyclone phenological recovery are scarce but have reported that trees do not produce fruits in the postdisturbance months [56,57]. Most plants regrow leaves in the postcyclone weeks [58–61]. However, damages may also trigger flowering in some species, in addition to sprouting and leaf growth [62].

In contrast to previous studies, the present study determined that the recovery of the Mearns fig is mainly directed toward fruit production: After the typhoon, all the individuals started producing figs in addition to new leaves. After defoliation, the plant vegetative system must be rebuilt to produce carbon again. Therefore, plants mostly use their carbon reserves, which must be replenished later [63]. Hence, the posttyphoon vegetative and reproductive regrowth of the Mearns fig is peculiar. Indeed, the plant lives in a harsh environment, which provides few nutrients, but these fig trees still produce syconia at the earliest.

**Fig 9:**
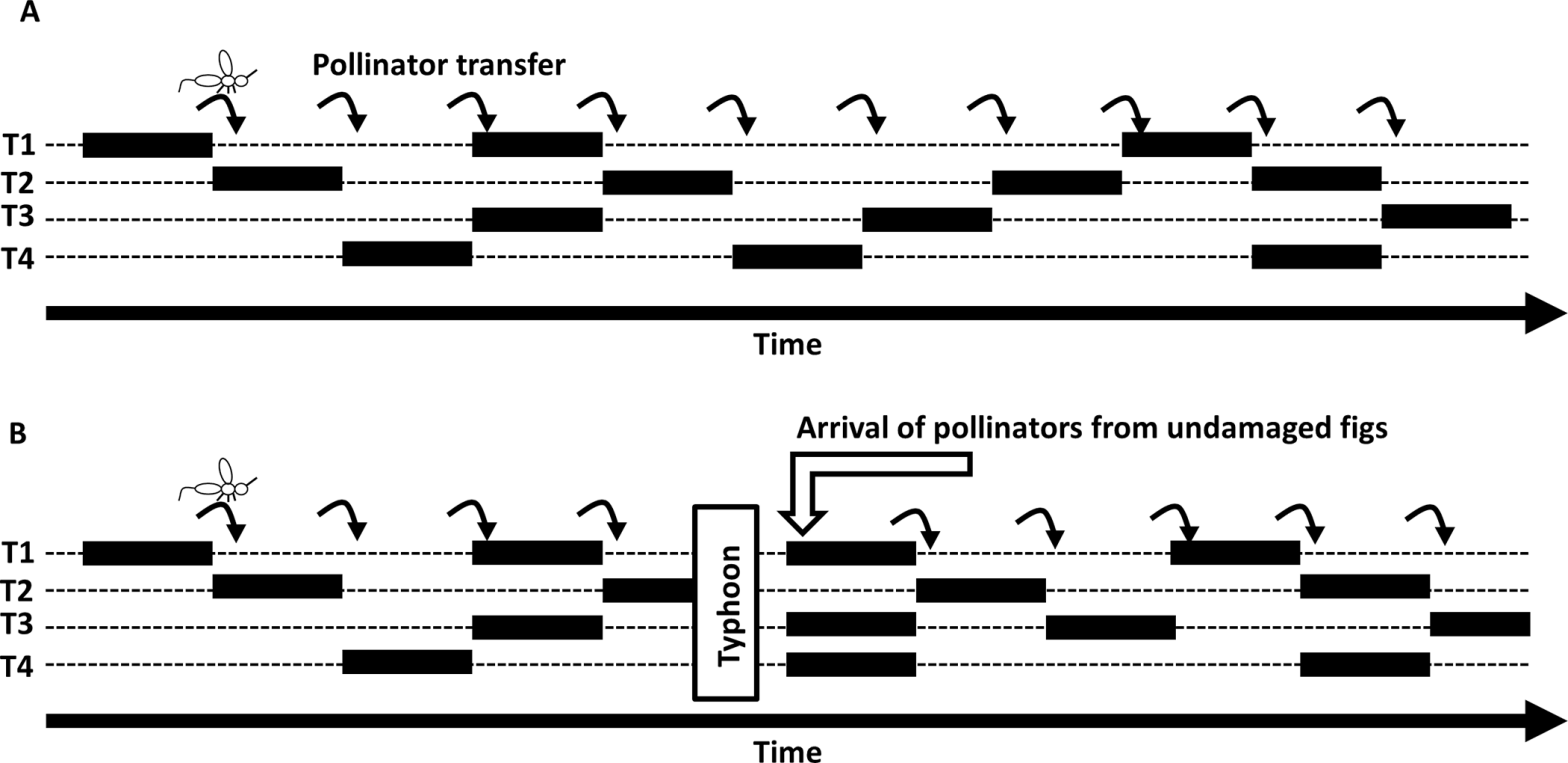
Schematization of the pollinator transfer within a fig population. In the case of an undisturbed population (A) and in the case of a typhoon destroying all the current crops of the population (B).

*Ficus* trees and their pollinators are bound together in an obligate mutualism with their associated pollinating wasp species, which lay eggs inside figs [34]. The lifespan of these tiny pollinators is up to a few days; therefore, the fig population cannot survive if the fig trees have the typical (mostly annual) reproductive phenology as do most angiosperm species. Many fig tree populations produce figs throughout the year [27,37]. This feature permits the pollinating fig wasps to find receptive figs in range at any time (Fig 9A). When drastic climatic events affect the population of fig trees, the trees lose all their figs and subsequently all the pollinators inside (Fig 9B). This phenomenon was reported after Typhoon Morakot in Southern Taiwan, but it was also reported during a severe drought in Borneo caused by El Niño [38]. Once the current crops are lost, the new crops are completely dependent on pollinating wasps from other populations to pollinate them and recolonize locally [38].

Climate change strongly affects the uplifted coral reef remnant ecosystem of the Hengchun Peninsula. The aboveground vegetation was withered by Typhoon Morakot, and the posttyphoon days were severely dry and witnessed warm foehn-like winds [52], which reduced the humidity. Plants living in such harsh, uncertain environments have been selected for their ability to survive withering typhoons; however, because of global climate change, extreme events, such as strong typhoons and droughts, occurred increasingly and more powerfully [15,25]. In the case of the Mearns fig, the recovery ability of coastal plants after extreme events could be affected by the increased frequency of typhoons since 2000 (from 3.3 to 5.7 [5]); in particular, after the complete withering of the local figs, pollinators from another population are required for recolonization. However, how can recolonization occur when the entire distribution area of the species is disturbed by typhoons during the same season? For example, during the 2016 Pacific typhoon season, the path of category 5 Super Typhoon Meranti was extremely close to the Hengchun Peninsula and Batanes Islands in September, and the enormous Typhoon Megi passed by the eastern coast of Taiwan, potentially affecting the eastern and Green Island Mearns fig populations. These two typhoons came in the range of most Mearns fig populations, possibly affecting the recovery ability of the whole fig and pollinating wasp populations.

Although the habitat of coastal uplifted coral reef remnants is harsh, various terrain anfractuosities would provide shelter for a few trees during typhoons, preventing some syconia and branches from being withered. Surviving C-phase syconia inhabited by pollinator larvae can be crucial for the local wasp population. However, with the increasing frequency of extreme events, the extinction risk of the local pollinated wasp population is increasing. Harrison et al. [64] reported withered fig trees with no syconia during the extreme drought following El Niño in Borneo in 1998, and the local wasp population had disappeared. Because the frequency of typhoons has increased only since 2000 [5], the effects of typhoons on the fig population are still new and must be closely monitored. For example, the posttyphoon tree mortality and pollination rates are major factors to be surveyed.

### Phenological sexual differentiation

After Typhoon Morakot, many aspects of leaf and syconium production were similar between male and female trees, but male trees had more pollinated syconia than did female trees, resulting in varying fig:leaf ratios. Syconium and leaf production can be linked in *Ficus* species such as *F*. *septica* from subgenus *Sycomorus* [65] and *F*. *tinctoria* from subgenus *Sycidium* [66]. However, the Mearns fig belongs to the subgenus *Ficus*, and contrastingly, leaf and syconium production are correlated in female trees but not in male trees. Moreover, female syconium production was correlated with rainfall. Syconium production is a carbon sink, particularly in female trees [67]. Female syconia are costlier to produce than male syconia. Therefore, female trees produce syconia in the best season of the year in many species [27,68,69] and also because they are not bound by the requirement of mutualism as are male trees [27]. Specifically, male trees have to produce frequently to maintain the pollinating wasp population (Fig 7). This constraint on male trees explains both the phenological difference with female trees and the rapid posttyphoon syconium production.

## Conclusion

The rapid posttyphoon recovery of the Mearns fig under the harsh conditions of the Hengchun Peninsula coast reveals that the species is particularly well adapted to its environment. However, because of the increasing frequency of strong typhoons and increasing sea level, the recovery ability of the Mearns fig may not be sufficient. The *Ficus* and its associated symbiotic insects may be at risk of local extinction. The entire distribution area of the Mearns fig has the same risks; therefore, the extinction risk extends to the entire variety. Because of climate change, the risk of extinction increases [70], and many species disappear even before being described [71]. The Mearns fig and its associated wasp species are an addition to the list of threatened species whose populations must be closely monitored.

## Supporting information

S1 Table Kolmogorov-Smirnov Z test between male and female productions of leaf and syconium. (PDF)

S2 Table Phenology raw data. (XLS)

S1 Fig Syconium crops of *Ficus pedunculosa* var. *mearnsii* for both genders in Frog Rock Trail during September 2009 to August 2011. Continuous lines represent full crops and dashed lines represent aborted crops.
(TIF)
