# Supplementary material for: Effects of climate change on a mutualistic coastal species: Recovery from typhoon damages and risks of population erosion
Source: PLoS One. 2017 Oct 26;12(10):e0186763. doi: 10.1371/journal.pone.0186763 (PMC5658060; doi:10.1371/journal.pone.0186763)
Supplement: S1 Table — (PDF) [file pone.0186763.s001.pdf]

S1 Table. Kolmogorov-Smirnov Z test between male and female productions of leaf and syconium.

	Fig					leaf				
Phases	A	B	C	D/E	Total	Tender	Mature	Yellow	Total	Fig/Leaf ratio
Z value	1.116	0.911	1.507	0.836	1.200	0.573	0.907	0.538	1.022	1.978
P value	0.166	0.378	<u>0.021</u>	0.487	0.112	0.897	0.384	0.935	0.247	<u>0.001</u>
