# Supplementary figures and images for: Effects of climate change on a mutualistic coastal species: Recovery from typhoon damages and risks of population erosion
Source: PLoS One. 2017 Oct 26;12(10):e0186763. doi: 10.1371/journal.pone.0186763 (PMC5658060; doi:10.1371/journal.pone.0186763)

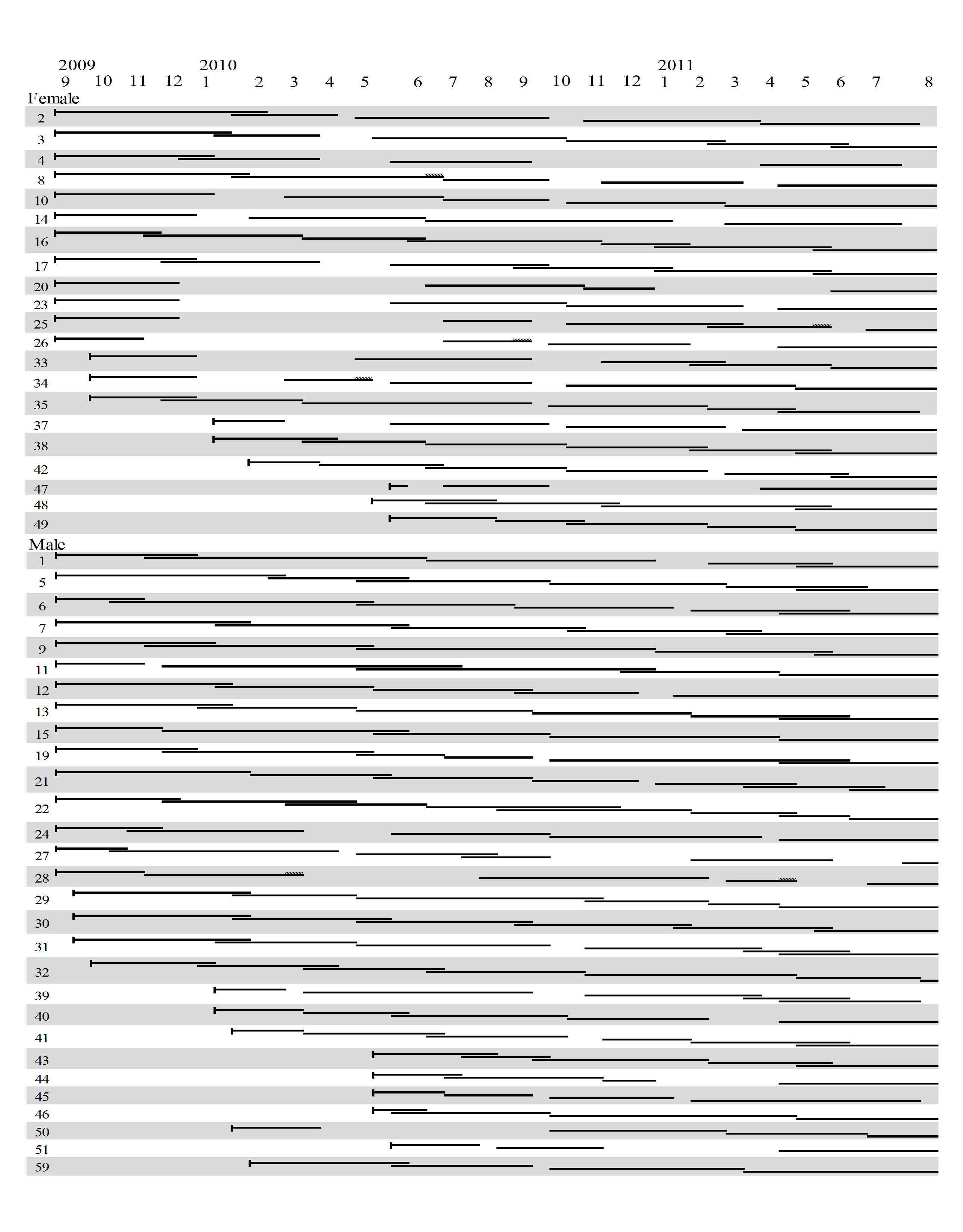

Supplement: S1 Fig — Continuous lines represent full crops and dashed lines represent aborted crops. (TIF) [file pone.0186763.s003.tif]
